# Molecular phylogenetic analysis of new *Entoloma rhodopolium*-related species in Japan and its identification method using PCR-RFLP

**DOI:** 10.1038/s41598-017-14466-x

**Published:** 2017-11-02

**Authors:** Kazunari Kondo, Kosuke Nakamura, Takumi Ishigaki, Kozue Sakata, Saemi Obitsu, Akio Noguchi, Nozomi Fukuda, Eiji Nagasawa, Reiko Teshima, Tomoko Nishimaki-Mogami

**Affiliations:** 1 Division of Biochemistry, National Institute of Health Sciences, 3-25-26 Tonomachi, Kawasaki-ku, Kawasaki, 210-9501 Japan; 2 The Tottori Mycological Institute 211 Kokoge, Tottori-shi, Tottori, 689-1125 Japan; 3 Pharmaceuticals and Medical Devices Agency 3-3-2 Kasumigaseki, Chiyoda-ku, Tokyo, 100-0013 Japan

## Abstract

Poisonous *Entoloma rhodopolium* and other similar species including edible *E. sarcopum* are morphologically diverse. People mistake poisonous species for edible species. Classification and the detection method of these species need to be defined. The morphological and phylogenetic studies have been reported in northern Europe. In Japan, the genetic study remains unsolved. Thus, phylogenetic analysis of *E. rhodopolium* was conducted using ITS and RPB2 sequences, and the result was compared with that of European species. Japanese *E. rhodopolium* was classified into three clades, none of which belonged to the true European *E. rhodopolium* and other known species. Three species were defined as new species. *Entoloma rhodopolium* clade-I (named *E. lacus*) was genetically close to but morphologically separated from *E. majaloides*. Clade-II (*E. subrhodopolium*) was classified to the same group as *E. sinuatum* and *E. subsinuatum*, but distinct from these species. Clade-III was segregated from known *Entoloma* species including *E. lupinum*, and named *E. pseudorhodopolium*. Based on the classification, a simple identification method PCR-RFLP was developed to discriminate between poisonous species and edible *E. sarcopum*, which is very similar in morphology. The study can help to clarify the taxonomy of complex *E. rhodopolium*-related species, and to prevent food poisoning.

## Introduction

The family *Entolomataceae* Kolt. & Pouzar, which belongs to the order *Agaricales*, has three main genera: *Rhodocybe* Maire, *Clitopilus* (Fr. ex Rabenh) P. Kumm. and *Entoloma* (Fr.) P. Kumm. s.l. Although most of the mushrooms in the family *Entolomataceae* are saprotrophic on soil and litter, some of them are ectomycorrhizal, such as *Entoloma rhodopolium* (Fr.) P. Kumm. and *Entoloma sinuatum* (Bull.) P. Kumm. The genus *Entoloma* s.l. is composed of more than 1,500 species and distributed worldwide^1–10^, and the genus is thought to include several subgenera that are recognized as separate genera by other authors^5,11,12^. Additionally, *Rhodocybe* and *Clitopilus* have been split into or segregated into several genera in contemporaneous classification^8,12^. Molecular phylogenetic study using three genetic makers RPB2, LSU and mtSSU together with spore morphology revealed that *Entoloma* s.l. is retained as one genus^8^. The study also pointed out that the smaller genera within *Entoloma* s.l. are either polyphyletic or make other genera paraphyletic. This indicates the importance of the molecular phylogenetic studies of *Entoloma* s.l., whose mushrooms vary in size, shape, and color, but either have pink gills or gills that become dusted with pink and pinkish or pinkish-brown spores in common. Among them, *E. rhodopolium* and other similar species, which is included in *Entoloma subgenus Rhodopolia* (Fr.) Noordel. ex Kokkonen^13^, are morphologically diverse and abundant in Japan. However, the phylogenetic study has not yet been reported, although only *E. rhodopolium* Tottori Er-1 (Accession number in NCBI, AB301602) is registered as *E. rhodopolium*. Whether it is identical to *E. rhodopolium* in Europe and North America remain unclear. In fact, the ITS sequence of *E. rhodopolium* Tottori Er-1 is distinct from that of true *E. rhodopolium* that Kokkonen reported as a neotype^13^. Therefore, a molecular phylogenetic study is needed to classify Japanese *E. rhodopolium* and compare European and North American species.

Many *Entoloma* species are reported poisonous such as *E. sinuatum* ^14^ in Europe and *E. rhodopolium* ^15,16^ in Japan, although some of them are edible such as *Entoloma abortivum* (Berk. & Curt.) Donk and *Entoloma sarcopum* Nagas. & Hongo (synonym *Rhodophyllus crassipes* (Imaz et Toki) Imaz et Hongo) (Nagasawa and Hongo 1999). *Entoloma sinuatum* is a notorious poisonous mushroom responsible for poisoning in Europe^17–19^. Morgado *et al*. have reported the species limits of *Entoloma* subg. *Entoloma*, showing that *E. sinuatum* distributes in Europe^10^. People wrongly identify *E. sinuatum* as edible sweetbread (*Clitopilus prunulus*) and St George’s (*Calocybe gambosa*) mushrooms in Europe^20^. In Japan, edible *E. sarcopum* resembles poisonous *E. rhodopolium*. Therefore, poisoning occurs many times every year. Ninety-five people suffered from mushroom poisoning last year according to the government statistics. To reduce human poisoning by these mushrooms, a new identification method is strongly needed. Identification of poisonous mushrooms was previously reported for four mushrooms, *Omphalotus japonicus* (Kawam.) Kirchm. & O. K. Mill., *E. rhodopolium* Tottori Er-1, *Tricholoma ustale* (Fr.) P. Kumm, and *Clitocybe acromelalga* by the real-time PCR method^21^. However, *E. rhodopolium* in the previous report may be different from true European *E. rhodopolium*. In addition, the study did not provide any information on genetic variations and nucleotide polymorphisms within each species.

Here we demonstrate the phylogenetic study of Japanese *E. rhodopolium* by comparing with European *Entoloma* species and the simple identification method using PCR-restriction fragment length polymorphism (RFLP) Our findings provide a new significant data on the taxonomy of complex *E. rhodopolium*-related species, and can help to reduce the cases of mushroom poisoning.

## Results

### Morphological observation

**Figure 1:**
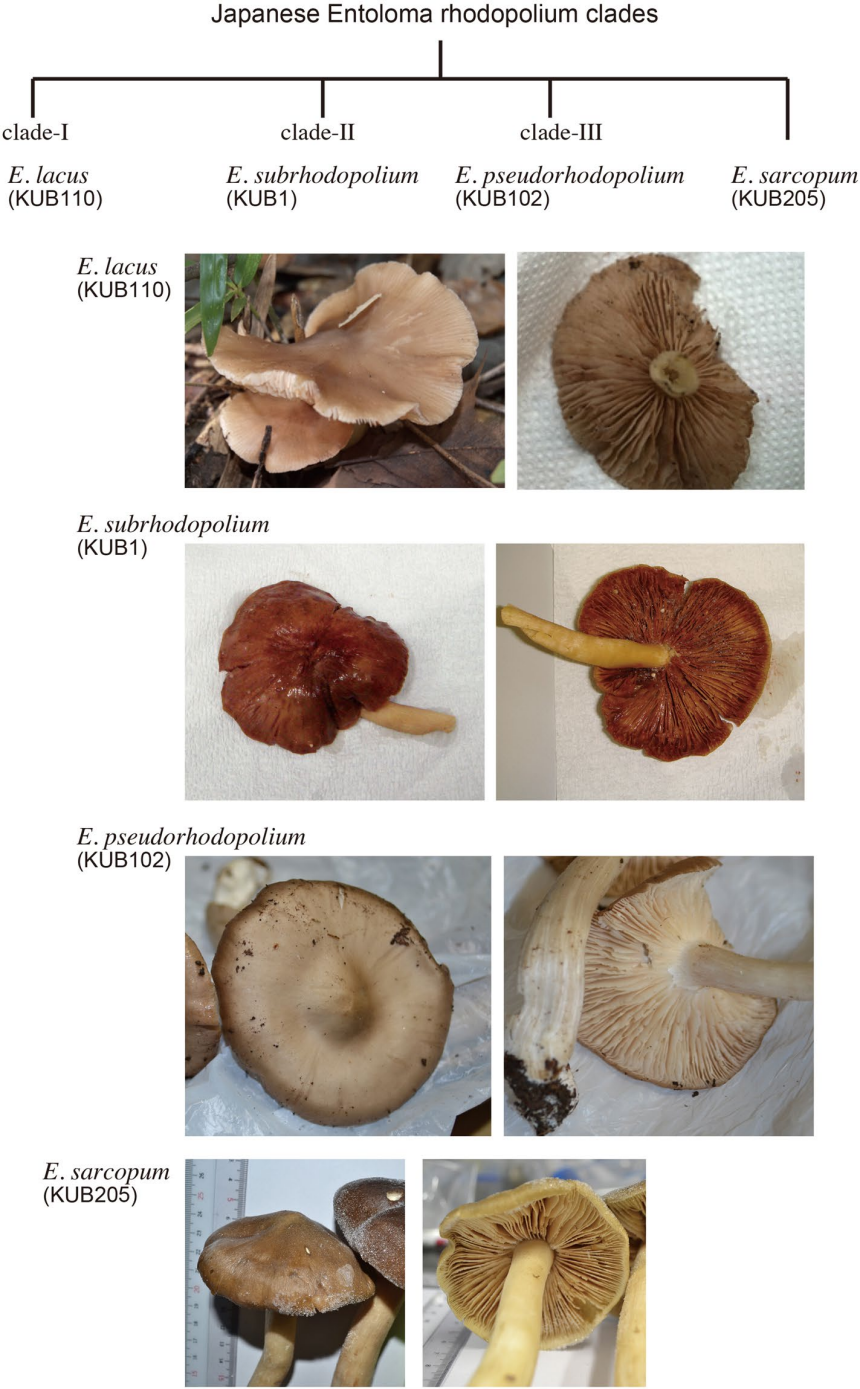
Pictures of Japanese *E. rhodopolium* clades. The clades-I, II, and –III were named *E. lacus*, *E. subrhodopolium*, and *E. pseudorhodopolium*, respectively. Holotypes are shown in this figure. *Entoloma sarcopum* is also shown for comparison.

**Figure 2:**
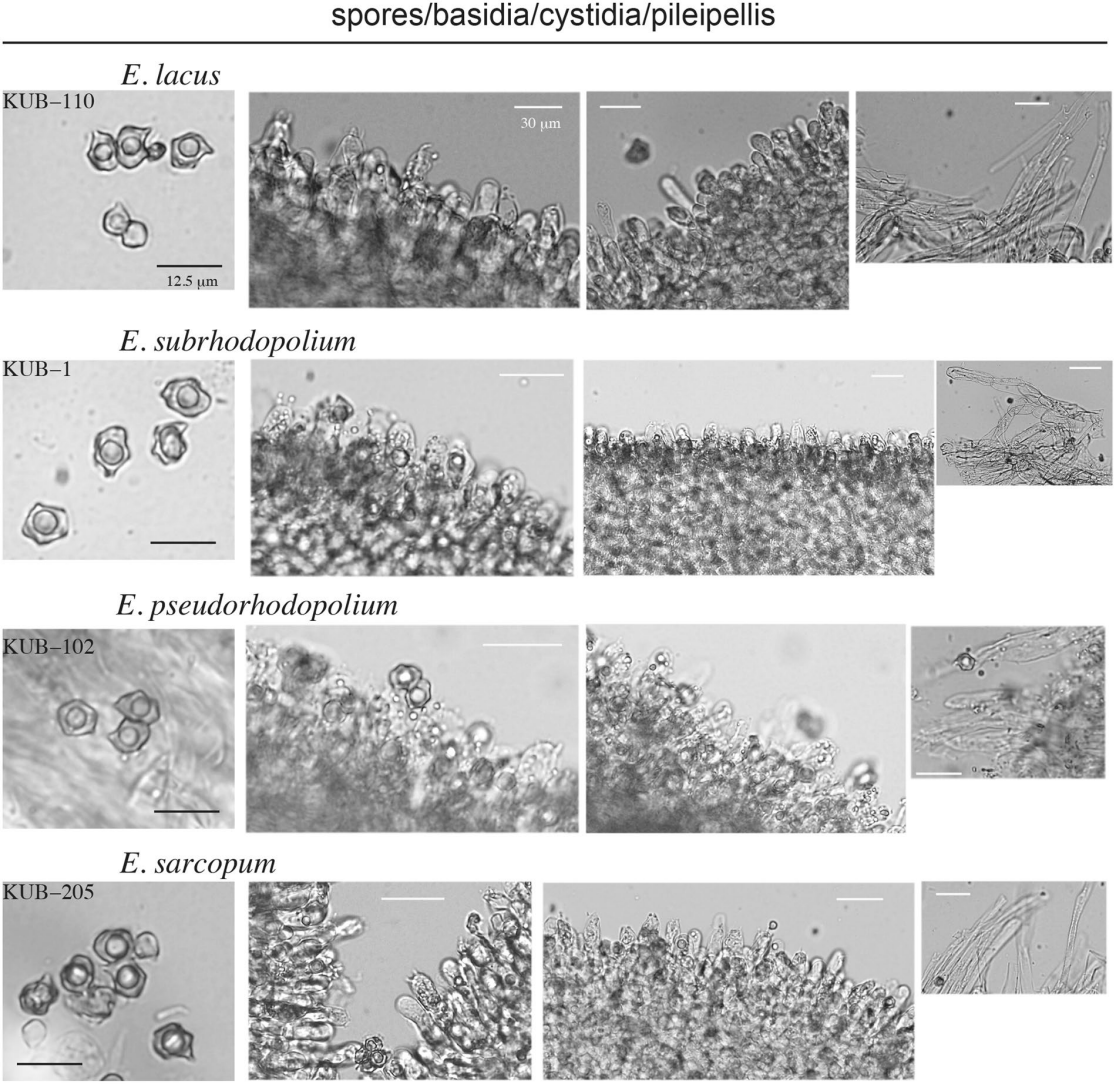
Images of spores, basidia, cystidia and pileipellis of *E.lacus,* *E. subrhodopolium*, *E. pseudorhodopolium*, and *E. sarcopum*. Images shows that spores 5–6 angles, basidia 2-, or 3-spored, cystidia the presence in *E. lacus*, the absence in *E. subrhodopolium* and *E. pseudorhodopolium*.

Characteristics of the mushrooms we considered *E. rhodopolium* in Japan are as follows: Pilei were 5 to 10 cm in diameter. Almost convex or flat to depressed. Pileus colors varied from grayish brown or yellowish gray to brown or reddish brown when matured, and were hygrophanous. Gills were mostly white and pinkish or brown when old. Stipes were 4 to 12 cm long and 5 to 15 mm wide, and mostly fragile but sometimes solid (Figs 1 and S1). Spores were heterodiametrical, and their average length × width and Q values were shown in the taxonomy section and Fig. S2. Basidia were 2 or 3-spored. Cystidia was not observed in many samples (Fig. 2). *Entoloma rhodopolium* belongs to *Entoloma* subgenus *Rhodopolia* (Fr.) Noordel.ex Kokkonen. The morphological characteristics of our *E. rhodopolium*, such as pileus, gill, stipe and spores, were similar to those described for *E. rhodopolium* in the previous report^13^. Typical *E. sarcopum* was collected whose characteristics were larger pileus (13 cm–18 cm) and longer and thicker stipe (15 cm>) than our *E. rhodopolium*, because a smaller size of *E. sarcopum* mushrooms are quite similar to our *E. rhodopolium*. The mushrooms we considered *E. sinuatum* was thicker and more solid than our *E. rhodopolium*. Representative photographs of our Japanese *E. rhodopolium* mushrooms are shown in Fig. 1.

### Phylogenetic analyses of ITS and RPB2 regions from Japanese *Entoloma rhodopolium*

**Figure 3:**
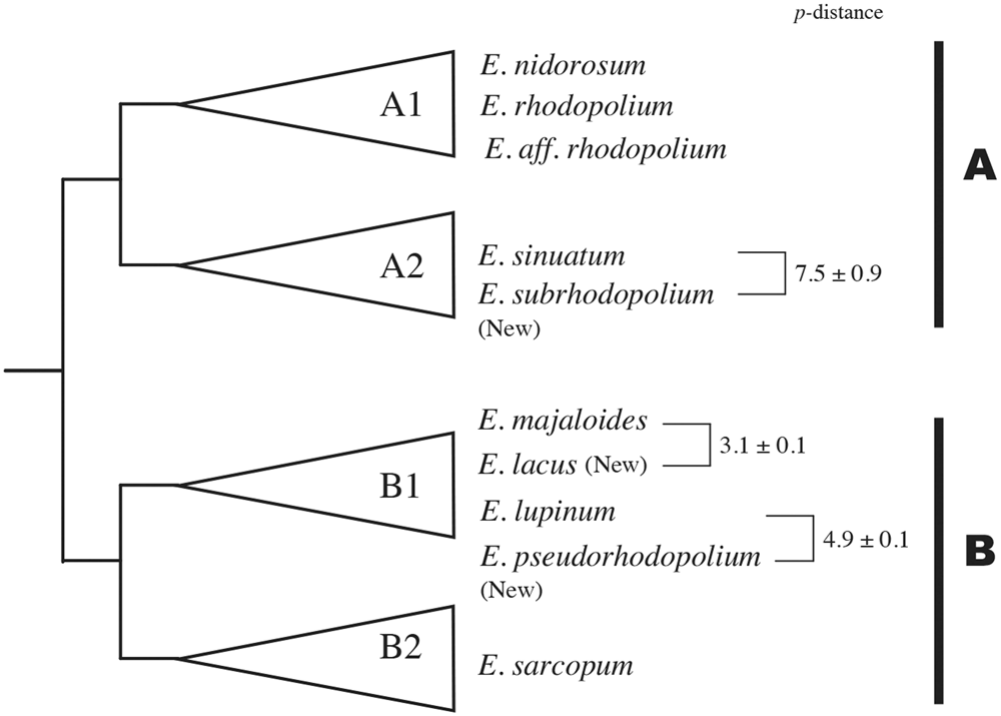
Layout of the phylogram obtained from maximum-likelihood analysis of ITS region using CLC genomic workbench software. *P*-distance between the specific species were shown in the figure. Detailed *p*-distances were described in the supplemental information (Fig. S3).

**Figure 4:**
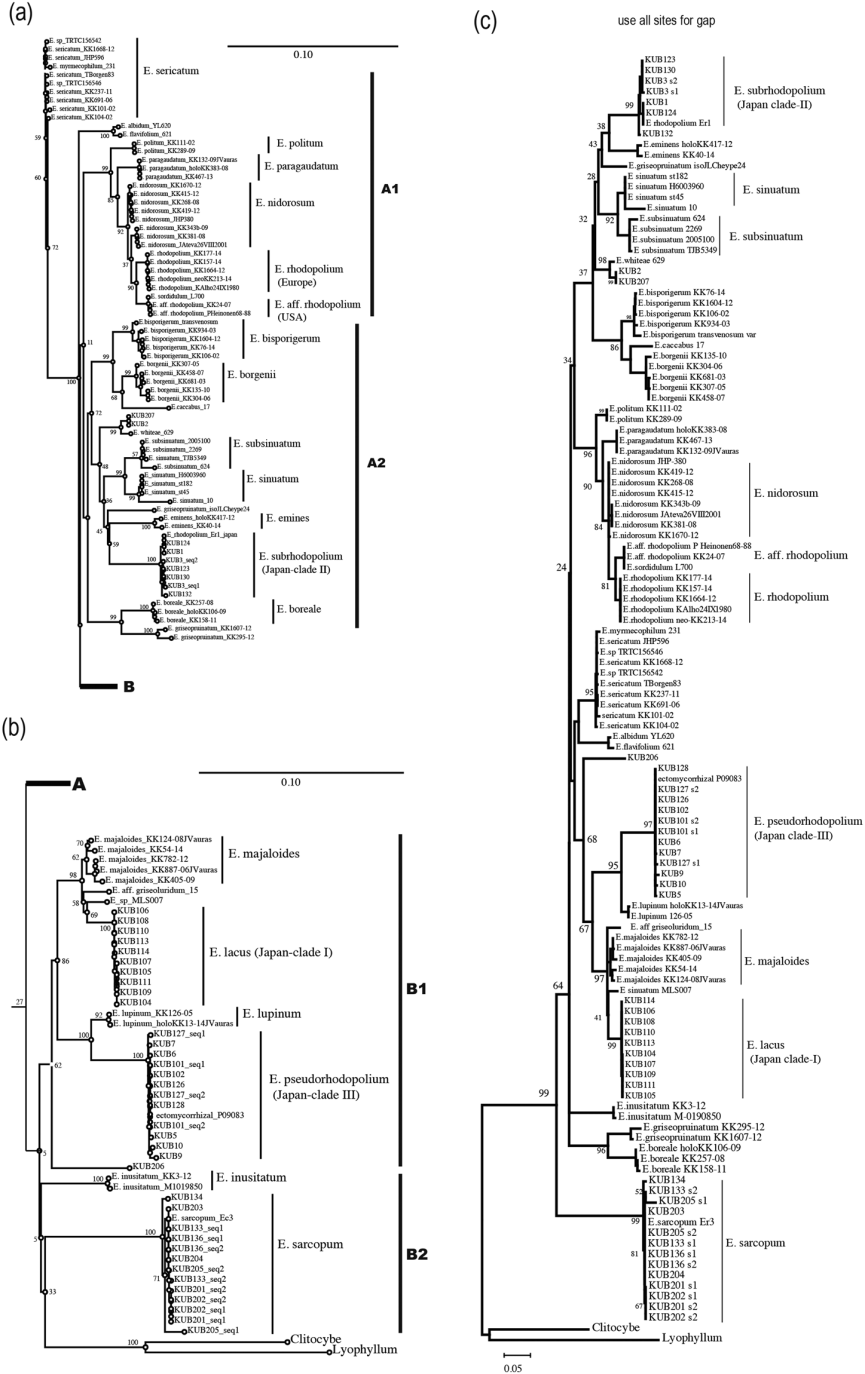
Phylogenetic trees from maximum likelihood analysis of ITS sequences. Bootstrap values are shown at each node as percentage (90 means 90% of replicates). (**a**) and (**b**) trees obtained from the analysis using CLC genomic workbench software. (**c**) A tree from MEGA7 analysis where all sites were used for gap/missing sites.

**Figure 5:**
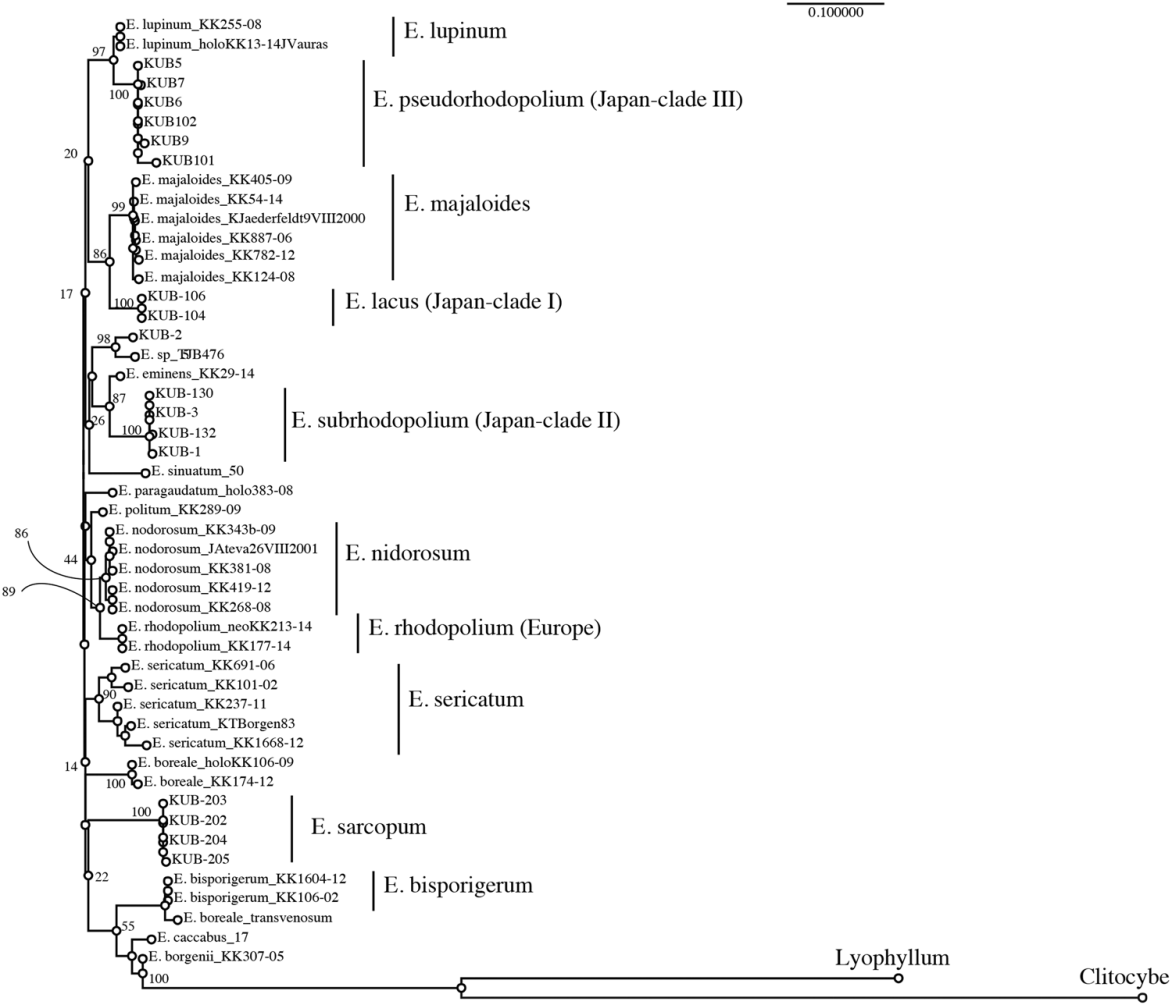
Phylogenetic tree from maximum likelihood analysis of RPB2 sequence. Bootstrap values (%) are shown at each node.

Samples and datasets used in this study are summarized in Tables S1 and S2. For molecular phylogenetic analyses, ITS and RPB2 regions were separately analyzed because RPB2 sequences were only obtained from some of our samples (named KUB) using CLC Genomics Workbench software. The layout of the phylogram obtained from the analysis of ITS region is shown in Fig. 3. Two large groups A and B were constructed, both of which include two subgroups A1, A2 and B1, B2, respectively. The phylogenetic analysis of ITS region revealed that our *E. rhodopolium* was separated into three clades (clade-I, -II, and –III). Interestingly, all three clades were segregated from European *E. rhodopolium* and North American *E*. aff. *rhodopolium* (Fig. 4a). *Entoloma nidorosum* was genetically close to European *E. rhodopolium* as reported by Kokkonen^13^. *E. rhodopolium* clade-II was clearly separated from A1 group including European *E. rhodopolium* and *E. nidorosum*, and was classified into A2 group containing *E. sinuatum* and *E. subsinuatum* (Fig. 4a). *Entoloma rhodopolium* clades-I and III were classified into B1 group on the phylogram (Fig. 3). The clade-I was genetically close to *E. majaloides*, whereas the clade-III was completely separated from other known species, although it is relatively close to *E. lupinum* (Fig. 4b). Edible *E. sarcopum* (B2 group) was distinct from the three *E. rhodopolium* clades. Next, RPB2 locus was analyzed. The result of RPB2 phylogenetic analysis was very similar to that of ITS analysis (Fig. 5). *Entoloma rhodopolium* mushrooms we collected were classified into the three clades, all of which were segregated from other known *Entoloma* species. The resolution power of ITS for molecular phylogenetic study was higher than that of RPB2 within *E. rhodopolium* and its related species. MEGA software was also used for the phylogenetic analysis of ITS region to compare our result with Kokkonen’s result, because MEGA can produce the same high quality data as PhyML or RAxML, and the recent analysis by Kokkonen was performed by the software. Three Japanese *E. rhodopolium* clades were distinctly separated each other. The analysis also supported the separation of *E. rhodopolium* clade-I from *E. majaloides*, the clade-II from *E. sinuatum*, *E. subsinuatum* and *E. eminens*, and the clade-III from *E. lupinum* (Fig. 4c). MEGA analysis also revealed that *p*-distance between *E. lacus* (clade-I) and *E. majaloides*, *E. subrhodopolium* (clade-II) and *E. sinuatum*, and *E. pseudorhodopolium* (clade-III) and *E. lupinum* were 3.1 ± 0.1, 7.9 ± 0.3, 4.9 ± 0.1 (%), respectively (Figs 3 and S3). Taken together, we here describe the three clades as new species. We name *Entoloma lacus, E. subrhodopolium*, and *E. pseudorhodopolium* for the *E. rhodopolium* clades-I, -II, and -III, respectively. We kept a word ‘*rhodopolium*’ for clades-II and -III, because these species have been considered *E. rhodopolium* in Japan for a long time.

### PCR-RFLP analysis

**Figure 6:**
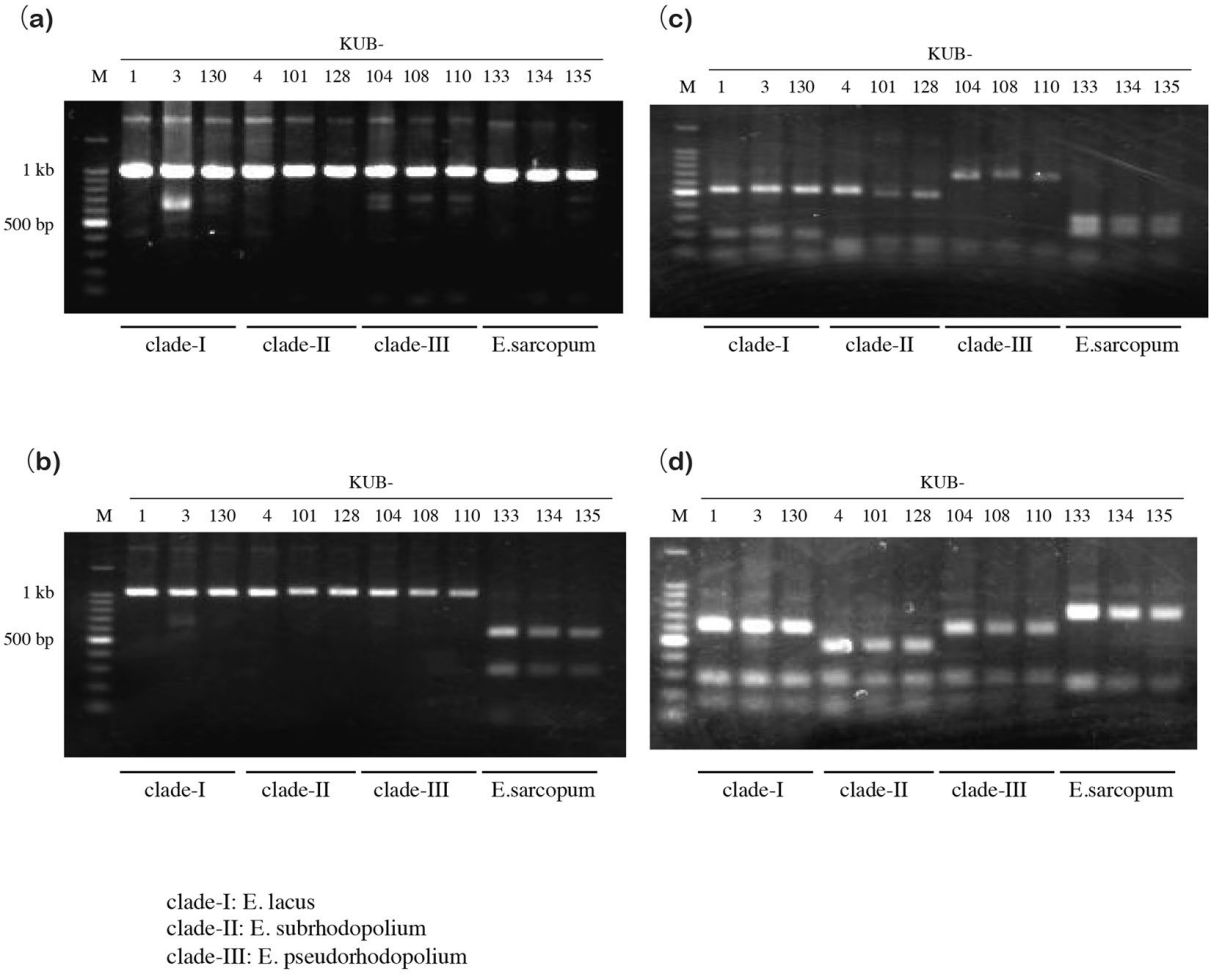
Results of PCR-RFLP using the ITS region. (**a**) PCR amplification of mushroom samples using universal primers. (**b**) *Msl*I (5′-CAYNN|NNRTG-3′) digested only edible *E. sarcopum* (5′-CATTG|GTATG-3′) mushrooms, but not Japanese *E. rhodopolium* clades (5′-CTYTG|RTATG-3′). The second nucleotide from 5′-terminus was ‘A’ in edible *E. sarcopum* and ‘T’ in poisonous *E. rhodopolium* clades. *MslI* digestion of PCR products in *A*. Edible *E. sarcopum* shows two clear bands and a smeared 80-bp band. (**c**) *Dde*I digestion of PCR products in *A*. *Entoloma sarcopum* shows three bands around 250 to 366 bp. *Entoloma rhodopolium* clade-I shows a distinct larger band at 690 bp, which is different from the other clades. (**d**) *Hinc*II/*Hae*III digestion, *Entoloma rhodopolium* clade-III shows a band at 502 bp, instead of 645 bp (*E. rhodopolium* clades-I and -II) and 802 bp (*E. sarcopum*).

Based on the results of the molecular phylogenetic studies, PCR-RFLP method was developed to discriminate poisonous Japanese *E. rhodopolium* clades and edible *E. sarcopum*. We selected *Msl*I, *Dde*I, and *Hinc*II/*Hae*III for RFLP after *in silico* simulation. Cleavage sites of the restriction enzymes in the ITS1-5.8S-ITS2 region is shown in Fig. S4. PCR reaction using primer pairs ITS1F/ITS4 produced a 1,075-bp band (Fig. 6a). When *Msl*I was used, the enzyme digested the PCR product from edible *E. sarcopum*, but not from *E. lacus* (*E. rhodopolium* clade-I)*, E. subrhodopolium* (*E. rhodopolium* clade-II), and *E. pseudorhodopolium* (*E. rhodopolium* clade-III) (Fig. 6b). *Dde*I digestion of *E. sarcopum* sample produced a distinct pattern showing 250-, 296-, and 366-bp bands along with weak bands at less than 100 bp. In contrast, *Entoloma lacus* (clade-I) gave a band at 690 bp and weak bands from 65 to 146 bp, which was distinct from the clades-II and III (Fig. 6c). However, *E. subrhodopolium* (clade-II) and *E. pseudorhodopolium* (clade-III) showed similar band patterns in *Dde*I digestion. Next, we performed *Hinc*II/*Hae*III double digestion. *Entoloma pseudorhodopolium* (clade-III) with a band at 502 bp was distinguished from *E. lacus* (clade-I, 645 bp), *E. subrhodopolium* (clade-II, 645 bp) and edible *E. sarcopum* (802 bp), as shown in Fig. 6d. Together, edible *E. sarcopum* and each three *E. rhodopolium* clades (*E. lacus, E. subrhodopolium, E. pseudorhodopolium*) were identified by the PCR-RFLP method.

### Short-length PCR-RFLP analysis

We examined a short-length PCR-RFLP analysis for cooked foods, in which the target sequence was located in the ITS2 region containing *Msl*I site. Three *E. sarcopum* samples and two Japanese *E. rhodopolium* samples were used in this experiment. The samples were treated with heat for 30 min and then treated with artificial gastric fluid for 30 min. The PCR products (214 bp length) of ITS2 region were subjected to *Msl*I digestion. Result of gel electrophoresis revealed that an intact band at 214 bp disappeared in *E. sarcopum* (KUB-201, -202, and -203), whereas the band was kept in our *E. subrhodopolium* (KUB-1) and *E. lacus* (KUB-104) as shown in Fig. 5S. This result shows that edible *E. sarcopum* and poisonous *E. rhodopolium* species can be detected and distinguished even in cooked food residues. Using quasi-mixed samples, we next tested whether a small portion of poisonous *E. subrhodopolium* could be detected in a large portion of edible mixed mushrooms. The PCR product was obtained from all samples (Fig. 6S, *left panel*). After *Msl*I digestion, samples that contained *E. subrhodopolium* (samples 2, 4, 5, and 7) retained the intact band, whereas the others (samples 1, 3, and 6) were digested by the enzyme (Fig. 6S, *right panel*). This indicates that even cooked and ingested food samples contaminated with a small amount of *E. subrhodopolium* can be detected.

**Figure 7:**
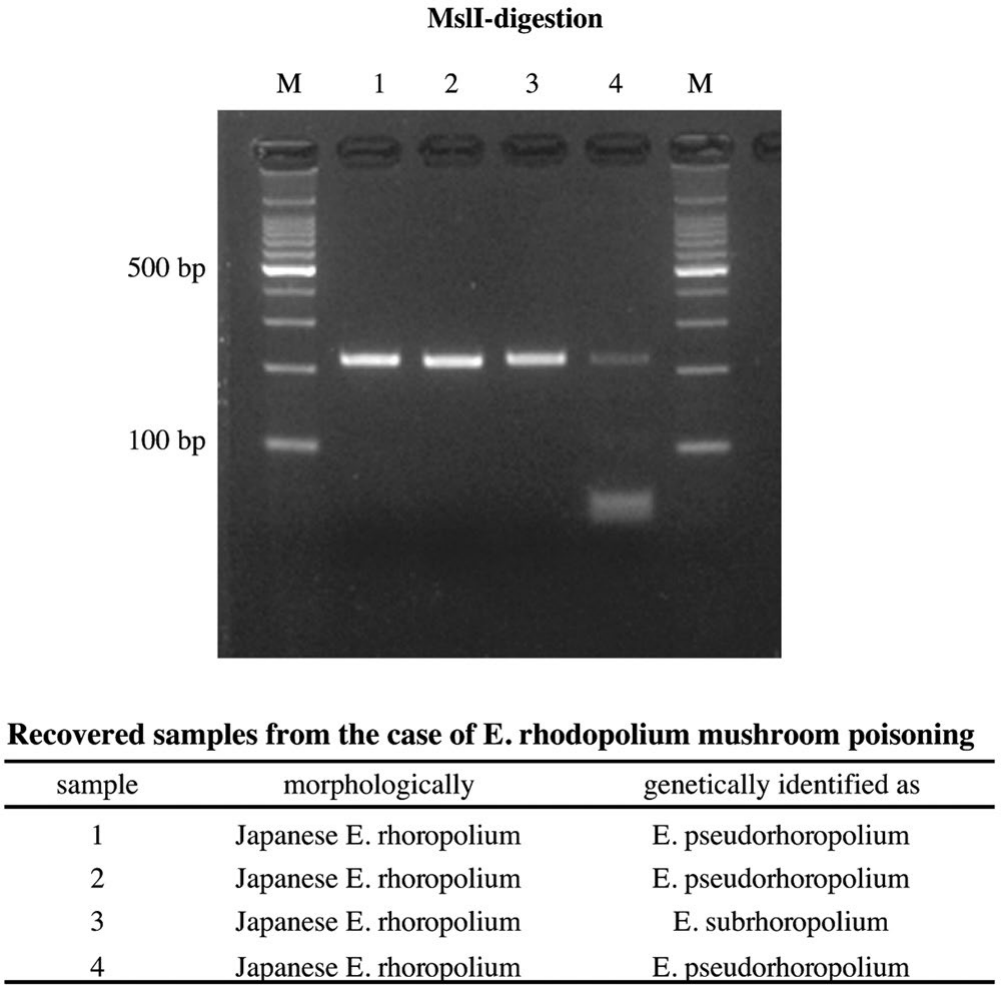
Analysis of recovered Japanese *E. rhodopolium* samples from the case of poisoning. Four samples recovered were examined to distinguish edible from three poisonous species by the short-PCR-RFLP. Classification of four samples were performed by PCR-RFLP and described in the table. Sample-4 considered mixed mushrooms.

To clarify the relationship between toxicity and Japanese *E. rhodopolium* clades (*E. lacus*, *E. subrhodopolium*, *E. subrhodopolium*), we analyzed mushroom samples collected from four cases of poisoning in the year 2015, all of which were morphologically identified as *E. rhodopolium*. Samples-1, 2, and 4 were *E. pseudorhodopolium* (clade-III), and sample-3 was *E. subrhodopolium* (clade-II). All samples were subjected to the PCR-RFLP analysis. The result shows that those clades are poisonous species (Fig. 7). We could not conclude that *E. lacus* is toxic because we haven’t collected it from poisoning cases. Further case studies need to be done.

**Taxonomy**

(Japan clade-I)

***Entoloma lacus*** Kondo, **sp. nov**. Figs 1, 2, 1S

MycoBank MB 818059

*Typification*: Holotype, Japan, Tokyo, Hachioji-city, Naganuma Park. 35° 38′ 19″ N, 139° 21′ 54″ E, Oct. 1 2011, K. Kondo, KUB-110 (**holotype** NIHS).

ITS_LN088049

*Entoloma lacus* grows in a moist mixed forest or wooded area near *Quercus serrata, Quercus acutissima*, and *Pinus densiflora* in October.

*Etymology*: lacus (Latin) refers to hollow stipe.

*Pileus* 6.5 cm in diameter (generally 4–8 cm), almost flat, light brown with striations in the marginal region, center of pileus slightly umbonate, smooth, slightly viscid when wet, margin undulate, hygrophanous. *Lamellae* pinkish brown, moderately crowded with free gills, adnate, edge even. *Stipe* 7.5 cm long (generally 5–10 cm), 0.6–0.8 cm wide (generally 0.6–1.1 cm), slightly light brown, fragile, hollow, fibrillose. *Context* in pileus concolorous, slightly pinkish brown, in stipe white to light brown. *Spores* 5–6-angled, 7.1-7.3-8.1/6.2-6.8-7.1 μm, Q = 1.0-1.1–1.2 (n = 20). *Basidia* 2- or 3-spored, 28–32 × 14–16 μm. (n = 7). *Cheilocystidia* occasional, 33–41 × 8–12 μm. (n = 5). cylindrical, straight to flexuous. *Pileipellis* hyphae hyaline, long and narrow, clavate. *Stipitipellis* terminal cells long clavate in bundles. *Clamps* present, *Smell* indistinct, slightly nitrous, unpleasant when well matured. Taste not bitter.

*Comments*: *E. lacus* may be a rare species and grow in a restricted area. Although it resembles *E. subrhodopolium* and *E. pseudorhodopolium* described below, *E. lacus* is distinctively smaller and has cystidia. *Entoloma lacus* is genetically close to, but distinctive from a stout species *E. majaloides*. *Entoloma lacus* does not have a darker umbo.

(Japan clade-II)

***Entoloma subrhodopolium*** Kondo & Nagasawa **sp. nov**. Figs 1, 2, 1S, 2S

MycoBank MB 818058.

*Typification*: Holotype, Japan, Yamagata, Chitoseyama, within 1 km of 38° 13′ 52″ N, 140° 21′ 45″ E, Oct. 2008, K. KONDO, KUB-1, (**holotype** NIHS).

ITS_LN088033, RPB2_LN148032

*Entoloma subrhodopolium* grows in a moist mixed forest near *Quercus serrata, Quercus acutissima*, and *Pinus densiflora* in late September to October.

*Etymology*: a word ‘*rhodopolium’* was kept because it has been considered *Entoloma rhodopolium* in Japan for a long time.

*Pileus* 9.5 cm in diameter (generally 4–10 cm), almost flat, slightly depressed in the center or broadly convex to applanate, brown, smooth, slightly viscid when wet, hygrophanous margin, no striations. *Lamellae* pinkish brown or brown, moderately crowded with short free gills. Sinuate (depressed around the stipe), edge even. *Stipe* 7.5 cm long (generally 6–10 cm), 1.0 cm wide (generally 0.8–1.4 cm), yellowish brown with white apex, fragile or sometimes stout, fibrillose. *Context* in pileus concolorous, white to slightly yellowish brown, in stipe white to light yellow. *Spores* 5-6-angled, 6.3-8.2-8.9/6.3-7.2-8.1 μm, Q = 1.0-1.1-1.2 (n = 20). *Basidia* 2- or 3-spored, 25–30 × 13–15 μm. (n = 5). *Cystidia* not observed. *Pileipellis* hyphae hyaline, relatively short and broad, or fusoid-ventricose, terminal cells somewhat broad cylindrical. *Stipitipellis* terminal cells long to short clavate in bundles. *Clamps* present, *Smell* indistinctive or nitrous and slightly unpleasaqnt odor when well matured. Taste not bitter.

*Comments*: *E. subrhodopolium* is genetically close to *E. sinuatum*, however, the latter is a stout and has a large ivory white pileus. *Entoloma subrhodopolium* is morphologically close to *E. pseudorhodopolium* but the latter has usually umbo at the center of pileus.

(Japan clade-III)

***Entoloma pseudorhodopolium*** Kondo & Nagasawa **sp. nov**. Figs 1, 2, 1S, 2S

MycoBank MB818057.

*Typification*: Holotype, Japan, Niigata, Tainai-city, Tainaidaira, within 2 km of 38° 1′ 22″ N, 139° 30′ 4″ E, Oct. 2011, K. KONDO, KUB-102 (**holotype** NIHS).

ITS_LN088042, RPB2_LN148040

*Entoloma pseudorhodopolium* grows in a moist mixed forest near *Quercus serrata, Quercus acutissima*, and *Pinus densiflora* in late September to October.

*Etymology*: a word ‘*rhodopolium*’ was kept because it has been considered *Entoloma rhodopolium* in Japan for a long time.

*Pileus* 5.8 cm in diameter (generally 4–12 cm), almost flat, slightly subconical in the center, umbonate, depressed, light brown, smooth, slightly viscid when wet, hygrophanous margin. *Lamellae* white but slightly brownish, moderately crowded with free gills. Sinuate (depressed around the stipe), edge uneven. *Stipe* 8.0 cm long (generally 5–10 cm), 0.8–1.2 cm wide (generally 0.6–1.2 cm), white to light brown with white apex, fragile or stout, fibrillose. *Context* in pileus concolorous, white to slightly brown, in stipe white to light yellow or brown. *Spores* 5-6-angled, 6.3-7.3-8.9/5.4-6.4-8.0 μm, Q = 1.0-1.2-1.4 (n = 18). *Basidia* 2- or 3-spored, 25–32 × 13–15 μm. (n = 5). *Cystidia* not observed. *Pileipellis* hyphae hyaline, relatively long and broad, terminal cells somewhat cylindrical, fusoid. *Stipitipellis* terminal cells long to short clavate in bundles. *Clamps* present, *Smell* indistinctive or nitrous and somewhat unpleasant odor when wet. *Taste* not bitter.

*Comments*: *E. pseudorhodopolium* is genetically close to *E. lupinum*, but the latter is stout with a grey-brown pileus, not with a cork or reddish-brown pileus like *E. pseudorhodopolium*. In addition, *E. lupinum* has cheliocyctidia. *Entoloma pseudorhodopolium* is morphologically similar to *E. subrhodopolium*. If the two species grow together, it would be rather difficult to distinguish each other, although E. subrhodopolium’s pileus is usually umbonate at the center.

*Habitat of the four Entoloma species described above* and below: they grow in broad-leaves trees of a mixed forest or wooden area, including *Quercus crispula* and *Quercus serrate* during September to October.

***Entoloma sarcopum*** Nagas. & Hongo. Reports of the Tottori Mycological Institute 37: 2 (1999)^22^.

MycoBank MB459971.

*Synonyms*: *Rhodophyllus crassipes* (Imazeki & Toki) Imazeki & Hongo, Journal of Japanese Botany 32: 146 (1957). Mycobank MB305221. *Entoloma crassipes* Imazeki & Toki, Bulletin of the Government Forest Experimental Station Meguro 67: 39 (1954). Mycobank MB282657.

KUB-205 (NIHS, a reference species in this article), Fig. 1.

ITS_LN088067, RPB2_LN148048

*Entoloma sarcopum* grows in a moist mixed forest near *Quercus serrata, Quercus acutissima*, and *Pinus densiflora* in late September to October.

Specimens (KUB-133-136) were used to identify the sequence of KUB-205. The specimens were considered *E. sarcopum* by comparing with holotype (TFM-F2947).

The species is usually large and strong but rarely small.

*Pileus* 8–10 cm in diameter, slightly subconical in the center, brown or darker brown, hygrophanous, fibrillose when dry, pruinose, slightly umbonate at the center, larger and thicker than *Entoloma rhodopolium*. *Lamellae* white to yellowish brown, moderately crowded, edge even to uneven. *Stipe* 10–15 cm long, 1.2–2.2 cm wide, white to light yellowish brown, solid, fibrillose. *Context* in pileus concolorous, white to slightly yellowish brown, in stipe white to light brown. *Spores* 5-6-angled, 8.0-8.2-8.9/6.3-7.0-8.0 μm, Q = 1.1-1.2-1.2 (n = 18). *Basidia* 2- or 3-spored, 25–27 × 10–13 μm. (n = 3). *Cystidia* not observed or rarely. *Pileipellis* hyphae hyaline, relatively long, clavate, terminal cells cylindrical. *Stipitipellis* terminal cells long clavate in bundles. *Clamps* present, *Smell* indistinctive or nitrous and somewhat unpleasant odor when wet. *Taste* strongly bitter even when cocked.

*Comments*: *E. sarcopum* grows in broad-leaves trees of a mixed forest or wooden area, including *Fagaceae Quercus crispula* and *Quercus serrate* during September to October together with *E. subrhodopolium and E. pseudorhodopolium*. *Entoloma sarcopum* has usually a larger pileus (13 cm–18 cm) and long thick stipe (15 cm>), however, when *E. sarcopum* grows smaller, it would be difficult to distinguish *E. sarcopum* from the latter two species.

## Discussion

In northern Europe, *E. rhodopolium* has been well studied and are now included in the subgenus *Rhodopolia* by Kokkonen. He reported boreal *Entoloma* species in Finland and introduced a neotype of *E. rhodopolium*. The aim of this study is to classify our Japanese *E. rhodopolium* clades and compare them with true European *E. rhodopolium*, and then to develop an easy-to-use identification method to prevent food poisoning caused by *E. rhodopolium* species. The molecular phylogenetic studies show that Japanese *E. rhodopolium* clades-I, -II, and III are distinct from European *E. rhodopolium*, North American *E*. aff. *rhodopolium*, *E. nidorosum* and other known *Entoloma* species. The result indicates our *E. rhodopolium* clades are new species. We named them *E. lacus, E. subrhodopolium* and *E. pseudorhodopolium*, respectively. this may help study the taxonomy of complex *Entoloma* species worldwide, indicating that there is morphological diversity within one species. To date, we have not collected true European *E. rhodopolium* in Japan. There have been no reports on mushroom poisoning by true *E*. *rhodopolium*. The fact raises a new question about which species are toxic among Japanese *E*. *rhodopolium* clades. Since last year, we analyzed mushroom samples recovered from the case of food poisoning, and found that at least *E. subrhodopolium* (Japanese *E. rhodopolium* clade-II) and *E. pseudorhodopolium* (clade-III) considered poisonous. Poisoning by *E. lacus* (clade-I) has not been reported. We collected *E. lacus* at one local area in the suburbs of Tokyo. *E. lacus* might be a minor species. As a result, we cannot tell whether or not this is edible or poisonous at this moment. KUB-2, 206, and 207 formed another clade, which might be new species. However, we did not define these species because we do not have whole fruit bodies to describe holotype after our extensive experiments.

Next, we show that the PCR-RFLP method can distinguish three Japanese *E. rhodopolium* clades (i.e. *E. lacus*, *E. subrhodopolium*, and *E. pseudorhodopolium*) from *E. sarcopum* when fresh samples were used. When this method is used before ingesting, it can reduce the risk of ingesting poisonous species. In cooked and ingested food residues, however, we could not distinguish between edible *E. sarcopum* and poisonous *E. lacus*, *E. subrhodopolium*, and *E. pseudorhodopolium* because of DNA degradation. To prevent mushroom poisoning, discrimination between edible and toxic mushrooms in food residues is more important and essential. Therefore, the short-length PCR-RFLP method was developed. Even samples boiled with heat and ingested with artificial gastric fluid were detected. In KUB-201, it retained a weak intact band, therefore, digestion may have not completed, or KUB-201 may have our *E. rhodopolium*. At any rate, poisonous mushrooms were not identified as edible mushrooms mistakenly. In addition, this method can detect a small amount (eg. 20 mg) of our *E. rhodopolium* mushrooms in a mixed edible mushroom sample (eg. 380 mg) that contained species such as *L. edodes, A. bisporus, G. frondosa, P. eryngii*, and *P. nameko*, which indicates the usefulness of short-length PCR-RFLP. We finally checked the sequences of European species using NCBI data sets and whether *Msl*I can digest European *E. rhodopolium*, *E*. aff. *rhodopolium*, *E. nidorosum*, *E. majaloides*, *E. inusitatum*, and *E. sericatum*. We found that they would give different band patterns each other. The four enzymes, *Msl*I, *Dde*I, and *Hinc*II/*Hae*III, are also useful for PCR-RFLP in European species.

In conclusion, we found three new *Entoloma rhodopolium*-related species by the molecular phylogenetic studies. Base on the studies, the identification method was developed using PCR-RFLP, which worked well. Our results may help to classify complex *Entoloma* species in the world, and to reduce food poisoning by *Entoloma* mushrooms.

## Methods

### Fungal sampling, DNA isolation, PCR, sequencing and dataset assembly

To examine genetic variations, we collected *E. rhodopolium*, *E. sinuatum* and *E. sarcopum* mushrooms from various regions in Japan, from Hokkaido (northern) to Shimane (southwestern) to cover genetic variations among locations (Table 1S). *Entoloma rhodopolium* were morphologically identified based on the comparison with the neotype reported by Kokkonen^13^ and our observations. Additionally, two samples that were considered *E. sinuatum* and nine of *E. sarcopum*, were collected. Genomic DNA was extracted using DNeasy Plant Mini Kit (Qiagen) or by the CTAB method following grinding in liquid nitrogen.

### PCR and alignment

Two markers, internal-transcribed spacers (ITS) and RNA polymerase subunit II (RPB2), were used. The sequencing primers for ITS were ITS-1F and ITS-4^23,24^. The primers for RPB2 were RPB2-6F and RPB2-7R for the first PCR, and RPB2-i6F and RPB2-i7R for the second nested PCR^8^. PCR amplification was performed in a 50 μL reaction volume: 1 μL primers (final 0.5–1 μM), 10 μL 10 × PCR buffer, 4 μL dNTP (200–400 μM), 5 μL DNA template (50 μg), 1 μL KOD-FX or KOD-FX neo (1.0U, Toyobo, Japan), and sterile MilliQ water. The PCR reaction conditions used for ITS were: 3 min initial incubation at 95 °C, followed by 45 cycles of 95 °C for 30 s, 55 °C for 30 s, and 72 °C for 1 min. The first PCR conditions for RPB2 were: 3 min initial incubation at 95 °C, followed by 30 cycles of 95 °C for 30 s, 61 °C for 30 s, and 72 °C for 1 min. The second nested PCR conditions for RPB2 were 3 min initial incubation at 95 °C, followed by 30 cycles of 95 °C for 30 s, 51 °C for 30 s, and 72 °C for 45 s. Sequences obtained were aligned using the MUSCLE^25,26^ multiple sequence alignment programs in CLC Genomics Workbench ver.8.5 (CLC Bio, Aarhus, Denmark) as described in the *Molecular phylogeny* section below. For alignment of these *Entoloma* species, datasets from our study and sequences from the NCBI database were used, and the sequences were adjusted to the same sequence length. ITS and RPB2 yielded alignment lengths of 926 and 621 bp, respectively. We collected 48 samples of *E. rhodopolium, E. sinuatum*, *and E. sarcopum*. From those samples, 37 ITS and 15 RPB2 sequences were obtained.

### Molecular phylogeny

Phylogenetic analyses were performed by the maximum likelihood method using a general-time-reversible model with a gamma distribution rate of variable sites in the CLC Genomics Workbench (ver.8.5). Bootstrap values (%) were calculated with 1,000 replicates, and a phylogenic tree was constructed. For the out-groups, two taxa were used; sequences of *Clitocybe dealbata* and *Lyophyllum leucophaeatum* were downloaded from NCBI. Phylogenetic trees were rooted by outgroups. For the in-group, a total of 37 *Entoloma* spp. were sequenced for ITS. Sequences of other *Entoloma* species were obtained from NCBI. All reference datasets are summarized in Tables 1S and 2S. When two sequences were obtained from a single mushroom (e.g., KUB-127), the sequences were designated as -seq 1 and -seq 2 (e.g., KUB-127-seq 1 and KUB-127-seq 2) in phylogenetic trees. KUB-127-seq 1 and -seq 2 were the same sequence except for a nine-nucleotide insertion or deletion (indel). Other KUB samples, such as KUB-3-seq 1 and–seq 2 also had the same sequence except for a one-nucleotide indel. Two sequences from a single sample were categorized within the same clade. MEGA7 software was also used to compare our data with the previous report. Phylogenetic analysis was performed by the maximum likelihood method using a general-time-reversible model with a gamma distribution rate of invariable sites. Gap/missing sites were used. Bootstrap values (%) were calculated with 500 replicates.

### Selection of restriction endonucleases and PCR-RFLP procedure

To select restriction endonucleases, *in silico* simulation was performed on the *In-silico* simulation of molecular biology experiments web site (http://insilico.ehu.es/), and proposed restriction endonuclease sites were evaluated using the Genetyx software (ver12, Genetyx Co., Japan). We selected four enzymes (*Msl*I, *Dde*I, *Hinc*II/*Hae*III) for PCR-RFLP to discriminate edible from poisonous mushrooms, which is the most important criterion for enzyme selection to identify poisoning mushrooms. Because of the genetic variations, *Msl*I, which recognizes CAYNN|NNRTG (CATTG|GTATG in *E. sarcopum*), was selected.

### PCR-RFLP analysis of ITS region

A quick DNA extraction method was used for the PCR-RFLP analysis. Briefly, mushrooms were washed with MilliQ water, and took an approximately 100-mg portion was placed in a 1.5-mL Eppendorf tube. The mushrooms were homogenated using a BioMasher II in 400 μL PrepMan Ultra Sample Preparation Reagent, and incubated at 100 C for 10 min. Then, samples were centrifuged at 13,000 × g for 2 min. The supernatant was used as PCR template for PCR-RFLP. PCR amplification for the ITS region was performed using BIOTAQ HS DNA polymerase (Bioline, London, UK) and primers, ITS1 and ITS4. PCR reaction conditions were the same as those in *Fungal sampling, DNA isolation, PCR, sequencing and dataset assembly* section. For restriction endonuclease reaction, purified PCR product was digested using *Msl*I, *Dde*I, or a combination of *Hae*III and *Hinc*II (10U per 1 μg PCR product; FastDigest, ThermoFisher Scientific, MA, USA) for 5 min to 30 min at 37 °C, and the digested product was run on a 3% agarose gel in TAE buffer.

### Short-length PCR-RFLP analysis

We applied short-length PCR-RFLP analysis to samples from cooked and/or ingested residues. A target sequence of 214 bp that included the *Msl*I site was amplified, and digested with the endonuclease to discriminate edible mushrooms from poisonous mushrooms. Primers for the short-length PCR-RFLP analysis were Short-F (5′-GCTCTTCTTAAATGCATTAGC-3′) and Short-R (5′-TCGCTTCGTCAACCTG-3′). Mushroom samples (>100 mg) were boiled for 30 min, washed, and then subjected to the artificial gastric fluid [the first fluid for disintegration test (Nacalai Tesque, Kyoto, Japan)] for 1 h at 37 °C. After washing the sample with MilliQ water two times, 500 μL PrepMan Ultra Sample Preparation Reagent was added to the residue before boiling for 10 min. The supernatant was used for PCR template after centrifugation. PCR product was directly digested with *Msl*I, and the resulting product was visualized on an agarose gel described above. To further distinguish a variety of edible mushrooms from poisonous *Entoloma rhodopolium*, we made quasi-mixed mushroom sample of *Lentinula edodes* (Berk.) Pegler (377 mg)*, Agaricus bisporus* (J.E. Lange) Imbach (1,043 mg), *Flammulina velutipes* (Curt.) Sing. (507 mg), *Hypsizygus marmoreus* (Peck) H.E. Bigelow (591 mg)*, Grifola frondosa* (Dicks.) Gray (377 mg)*, Pleurotus eryngii* (DC.) Quél. (661 mg), and *Pholiota nameko* (T. Ito) S. Ito & S. Imai (*Pholiota microspora*) (985 mg). All of these edible mushrooms were cultured on mushroom beds purchased from supermarket.

## Electronic supplementary material

supplemental figures
