# Supplementary material for: Molecular phylogenetic analysis of new Entoloma rhodopolium-related species in Japan and its identification method using PCR-RFLP
Source: Sci Rep. 2017 Nov 2;7:14942. doi: 10.1038/s41598-017-14466-x (PMC5668239; doi:10.1038/s41598-017-14466-x)
Supplement: Supplementary file 1 — supplemental figures [file 41598_2017_14466_MOESM1_ESM.pdf]

(Supplemental Information)

**Molecular phylogenetic analysis of new *Entoloma rhodopolium*-related species in Japan and its identification method using PCR-RFLP.**

Kazunari Kondo[1,*], Kosuke Nakamura[1], Takumi Ishigaki[1], Kozue Sakata[1], Saemi Obitsu[1], Akio Noguchi[1], Nozomi Fukuda[1], Eiji Nagasawa[2], Reiko Teshima[3], Tomoko Nishimaki-Mogami[1]

[1,] *Division of Biochemistry, National Institute of Health Sciences*

1-18-1 *Kamiyoga, Setagaya, Tokyo* 158-0098, *Japan*

[2,] *The Tottori Mycological Institute*

211 *Kokoge, Tottori-shi, Tottori* 689-1125 *Japan*

[3,] *Pharmaceuticals and Medical Devices Agency*

3-3-2 *Kasumigaseki, Chiyoda-ku*, *Tokyo* 100-0013 *Japan*

*, Corresponding author. Current address: Division of Biochemistry, National Institute of Health Sciences, 1-18-1 Kamiyoga, Setagaya, Tokyo 1588501, Japan.

Tel.:+81-3-3700-9478, Fax.: +81-3-3707-6950.

**Table 1S** Mushroom samples collected in this study.

| sample name | | location | year | morphologically | Accession # | |
|---|---|---|---|---|---|---|
| | | | | | **ITS** | **RPB2** |
| KUB 1 | frozen | Yamagata | 2010 | *Entoloma rhodopolium* | LC088033 | LC148032 |
| KUB 2 | frozen | Yamagata | 2010 | *Entoloma rhodopolium* | LC088034 | LC148033 |
| KUB 3 | freeze-dried | Yamagata | 2010 | *Entoloma rhodopolium* | LC088035 | LC148034 |
| KUB 4 | freeze-dried | Shimane | 2008 | *Entoloma rhodopolium* | — | — |
| KUB 5 | freeze-dried | Shimane | 2008 | *Entoloma rhodopolium* | LC088036 | LC148035 |
| KUB 6 | freeze-dried | Shimane | 2008 | *Entoloma rhodopolium* | LC088037 | LC148036 |
| KUB 7 | freeze-dried | Shimane | 2008 | *Entoloma rhodopolium* | LC088038 | LC148037 |
| KUB 8 | freeze-dried | Shimane | 2008 | *Entoloma rhodopolium* | — | — |
| KUB 9 | freeze-dried | Shimane | 2008 | *Entoloma rhodopolium* | LC088039 | LC148038 |
| KUB 10 | freeze-dried | Shimane | 2008 | *Entoloma rhodopolium* | LC088040 | — |
| KUB 11 | freeze-dried | Shimane | 2008 | *Entoloma rhodopolium* | — | — |
| KUB 101 | frozen | Niigata | 2011 | *Entoloma rhodopolium* | LC088041 | LC148039 |
| KUB 102 | frozen | Niigata | 2011 | *Entoloma rhodopolium* | LC088042 | LC148040 |
| KUB 103 | frozen | Hokkaido | 2011 | *Entoloma rhodopolium* | — | — |
| KUB 104 | frozen | Tokyo | 2011 | *Entoloma rhodopolium* | LC088043 | LC148041 |
| KUB 105 | frozen | Tokyo | 2011 | *Entoloma rhodopolium* | LC088044 | — |
| KUB 106 | frozen | Tokyo | 2011 | *Entoloma rhodopolium* | LC088045 | LC148042 |
| KUB 107 | frozen | Tokyo | 2011 | *Entoloma rhodopolium* | LC088046 | — |
| KUB 108 | frozen | Tokyo | 2011 | *Entoloma rhodopolium* | LC088047 | — |
| KUB 109 | frozen | Tokyo | 2011 | *Entoloma rhodopolium* | LC088048 | — |
| KUB 110 | frozen | Tokyo | 2011 | *Entoloma rhodopolium* | LC088049 | — |
| KUB 111 | frozen | Tokyo | 2011 | *Entoloma rhodopolium* | LC088050 | — |
| KUB 112 | frozen | Tokyo | 2011 | *Entoloma rhodopolium* | — | — |
| KUB 113 | frozen | Tokyo | 2011 | *Entoloma rhodopolium* | LC088051 | — |
| KUB 114 | frozen | Tokyo | 2011 | *Entoloma rhodopolium* | LC088052 | — |
| KUB 121 | dried, specimen | Tottori | 2007 | *Entoloma rhodopolium* | — | — |
| KUB 122 | dried, specimen | Tottori | 2007 | *Entoloma rhodopolium* | — | — |
| KUB 123 | dried, specimen | Tottori | 2007 | *Entoloma rhodopolium* | LC088053 | — |
| KUB 124 | dried, specimen | Tottori | 2007 | *Entoloma rhodopolium* | LC088054 | — |
| KUB 125 | dried, specimen | Okayama | 2008 | *Entoloma rhodopolium* | — | — |
| KUB 126 | dried, specimen | Tottori | 2008 | *Entoloma rhodopolium* | LC088055 | — |
| KUB 127 | dried, specimen | Tottori | 2008 | *Entoloma rhodopolium* | LC088056 | — |
| KUB 128 | dried, specimen | Tottori | 2008 | *Entoloma rhodopolium* | LC088057 | — |
| KUB 129 | dried, specimen | Tottori | 2008 | *Entoloma rhodopolium* | — | — |
| KUB 130 | dried, specimen | Tottori | 2010 | *Entoloma rhodopolium* | LC088058 | LC148043 |
| KUB 131 | dried, specimen | Tottori | 2010 | *Entoloma rhodopolium* | — | — |
| KUB 132 | dried, specimen | Tottori | 2008 | *Entoloma rhodopolium* | LC088059 | LC148044 |
| KUB 133 | dried, specimen | Tottori | 2008 | *Entoloma sarcopum* | LC088060 | — |
| KUB 134 | dried, specimen | Tottori | 2010 | *Entoloma sarcopum* | LC088061 | — |
| KUB 135 | dried, specimen | Tottori | 2009 | *Entoloma sarcopum* | — | — |
| KUB 136 | dried, specimen | Tottori | 2008 | *Entoloma sarcopum* | LC088062 | — |
| KUB 201 | frozen | Yamagata | 2012 | *Entoloma sarcopum* | LC088063 | — |
| KUB 202 | frozen | Yamagata | 2012 | *Entoloma sarcopum* | LC088064 | LC148045 |
| KUB 203 | frozen | Fukushima | 2012 | *Entoloma sarcopum* | LC088065 | LC148046 |
| KUB 204 | frozen | Tochigi | 2010 | *Entoloma sarcopum* | LC088066 | LC148047 |
| KUB 205 | frozen | Tochigi | 2010 | *Entoloma sarcopum* | LC088067 | LC148048 |
| KUB 206 | frozen | Yamagata | 2012 | *Entoloma sinuatum?* | LC088068 | — |
| KUB 207 | frozen | Hokaido | 2012 | *Entoloma sinuatum?* | LC088069 | — |

(-), sequences were not obtained

| **Table 2S** Datasets used for molecular phylogenic study. | | |
|---|---|---|
| **Data name** | **Accession #** | |
| | ITS | RPB2 |
| *Entoloma bisporigerum* KK76-14 | LN850532 | — |
| *Entoloma bisporigerum* KK1604-12 | LN850533 | LN850681 |
| *Entoloma bisporigerum* KK106-02 | LN850534 | LN850682 |
| *Entoloma bisporigerum* KK934-03 | LN850535 | — |
| *Entoloma bisporigerum* transvenosum var | LN850538 | LN850683 |
| *Entoloma borgenii* KK135-10 | LN850522 | — |
| *Entoloma borgenii* KK304-06 | LN850520 | — |
| *Entoloma borgenii* KK681-03 | LN850521 | — |
| *Entoloma borgenii* KK307-05 | LN850519 | LN850680 |
| *Entoloma borgenii* KK458-07 | LN850523 | — |
| *Entoloma boreale* holoKK106-09 | LN850624 | LN850697 |
| *Entoloma boreale* KK257-08 | LN850622 | — |
| *Entoloma boreale* KK158-11 | LN850623 | — |
| *Entoloma eminens* holoKK417-12 | LN850584 | — |
| *Entoloma eminens* KK40-14 | LN850583 | — |
| *Entoloma griseopruinatum* KK295-12 | LN850590 | — |
| *Entoloma griseopruinatum* KK1607-12 | LN850588 | — |
| *Entoloma insitatum* KK3-12 | LN850542 | — |
| *Entoloma insitatum* topoM.Enderle6 | LN850543 | — |
| *Entoloma lupinum* holoKK13-14JVauras | LN850570 | LN850695 |
| *Entoloma lupinum* KK126-05 | LN850569 | — |
| *Entoloma majaloides* KK782-12 | LN850478 | LN850654 |
| *Entoloma majaloides* KK887-06JVauras | LN850479 | LN850655 |
| *Entoloma majaloides* KK405-09 | LN850482 | LN850660 |
| *Entoloma majaloides* KK54-14 | LN850488 | LN850667 |
| *Entoloma majaloides* KK124-08JVauras | LN850483 | LN850662 |
| *Entoloma nidorosum* KK268-08 | LN850501 | LN850672 |
| *Entoloma nidorosum* KK419-12 | LN850503 | LN850673 |
| *Entoloma nidorosum* KK1670-12 | LN850504 | LN850671 |
| *Entoloma nidorosum* KK415-12 | LN850502 | — |
| *Entoloma nidorosum* JHP380 | LN850509 | — |
| *Entoloma nidorosum* KK343b-09 | LN850505 | LN850675 |
| *Entoloma nidorosum* KK381-08 | LN850506 | LN850674 |
| *Entoloma nidorosum* J.Ateva26 | LN850507 | LN850676 |
| *Entoloma rhodopolium* KK157-14 | LN850496 | — |
| *Entoloma rhodopolium* KK1664-12 | LN850497 | LN850705 |
| *Entoloma rhodopolium* neoKK213-14 | LN850494 | LN850669 |
| *Entoloma rhodopolium* K.Alho24 | LN850498 | — |
| *Entoloma rhodopolium* KK177-14 | LN850495 | LN850670 |
| *Entoloma* aff. *rhodopolium* KK24-07 | LN850499 | — |
| *Entoloma* aff. *rhodopolium* PH | LN850500 | — |
| *Entoloma rhodopolium* Er-1 | AB301602 | — |
| *Entoloma sarcopum* EC-3 | AB301603 | — |
| *Entoloma sericatum* KK101-02 | LN850446 | LN850637 |
| *Entoloma sericatum* KK691-06 | LN850452 | LN850635 |
| *Entoloma sericatum* KK691-06 | LN850452 | LN850635 |
| *Entoloma sinuatum* st45 | KC710108 | — |
| *Entoloma sinuatum* st182 | KC710116 | — |
| *Entoloma sinuatum* H6003960 | GU373512 | — |
| *Entoloma sinuatum* TJB5349 | DQ486700 | — |
| *Entoloma sp* MLS007 | GQ397994 | — |
| uncultured ectomycorrhizal fungus | AB587746 | — |
| *Clitocybe dealbata* 11212 | JF907804 | DQ825407 |
| *Lyophyllum leucopaeatum* Hae251-97 | AF357032 | DQ367434 |

clade-I

*E. lacus* KUB-108

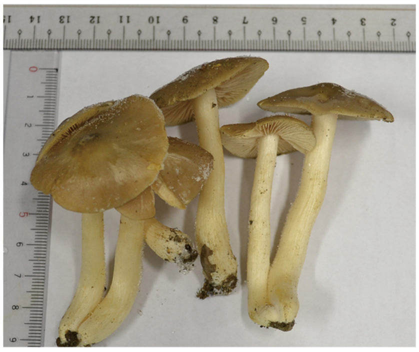

clade-II

*E. subrhodopolium* KUB-130

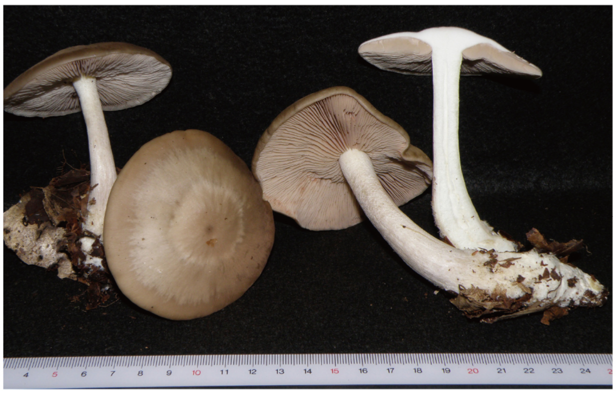

*E. pseudorhodopolium* clade-III

KUB-7

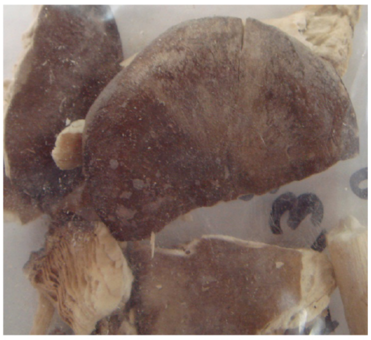

KUB-8

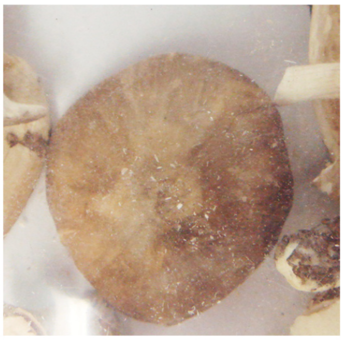

**Figure 1S.** Photographs of other samples from Japanese Entoloma rhodopolium clades.

*E. subrhodopolium*

clade-II

KUB-123

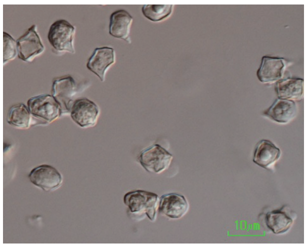

spore (mm)

length: 7.5-8.6-9.6
width: 6.0-6.4-7.2
Q = 1.3-1.3-1.5 (n=25)

KUB-124

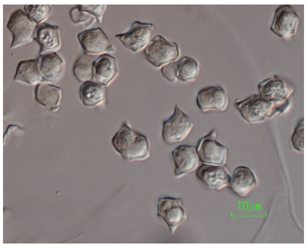

spore (mm)

length: 7.5-8.6-9.6
width: 6.0-6.4-7.2
Q = 1.2-1.3-1.5 (n=21)

*E. pseudorhodopolium*

clade-III

KUB-126

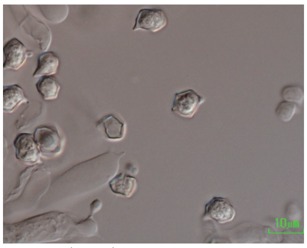

spore (mm)

length: 7.5-8.5-10.0
width: 6.0-6.9-7.5
Q = 1.1-1.2-1.5 (n=20)

KUB-127

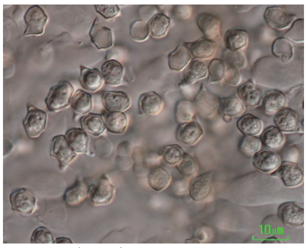

spore (mm)

length: 7.8-9.6-12.0
width: 6.0-7.2-9.3
Q = 1.1-1.3-1.7 (n=40)

(min-Ave-max)

**Figure 2S.** Photographs of spores from Japanese Entoloma rhodopolium clades-II and III.

## *p*-distance between known species

(a)

| | | *E.sinuatum* | | | *E.subsunuatum* | | | |
|---|---|---|---|---|---|---|---|---|
| | | 1 | 2 | 3 | 4 | 5 | 6 | 7 |
| 1 | E_sinuatum_st45 | | | | | | | |
| 2 | E_sinuatum_st182 | 0.000 | | | | | | |
| 3 | E_sinuatum_H6003960 | 0.000 | 0.000 | | | | | |
| 4 | subsinuatum_624 | 0.037 | 0.037 | 0.037 | | | | |
| 5 | subsinuatum _2269 | 0.026 | 0.026 | 0.026 | 0.011 | | | |
| 6 | subsinuatum_2005100 | 0.025 | 0.025 | 0.025 | 0.011 | 0.000 | | |
| 7 | E_subsinuatum_TJB5349 | 0.029 | 0.029 | 0.029 | 0.014 | 0.002 | 0.002 | |

2.9 ± 0.5 (%)
(sinuatum vs subsinuatum)

| | | *E.nidorosum* | | | | | | | | *E.rhodopolium* | | | | |
|---|---|---|---|---|---|---|---|---|---|---|---|---|---|---|
| | | 1 | 2 | 3 | 4 | 5 | 6 | 7 | 8 | 9 | 10 | 11 | 12 | 13 |
| 1 | nidorosum_JAteva26VIII2001 | | | | | | | | | | | | | |
| 2 | nidorosum_KK343b-09 | 0.000 | | | | | | | | | | | | |
| 3 | nidorosum_KK381-08 | 0.000 | 0.000 | | | | | | | | | | | |
| 4 | nidorosum_JHP-380 | 0.004 | 0.004 | 0.004 | | | | | | | | | | |
| 5 | nidorosum_KK1670-12 | 0.007 | 0.007 | 0.007 | 0.001 | | | | | | | | | |
| 6 | nidorosum_KK415-12 | 0.005 | 0.005 | 0.005 | 0.000 | 0.001 | | | | | | | | |
| 7 | nidorosum_KK268-08 | 0.005 | 0.005 | 0.005 | 0.000 | 0.001 | 0.000 | | | | | | | |
| 8 | nidorosum_KK419-12 | 0.005 | 0.005 | 0.005 | 0.000 | 0.001 | 0.000 | 0.000 | | | | | | |
| 9 | rhodopolium_KK177-14 | 0.015 | 0.015 | 0.015 | 0.016 | 0.019 | 0.017 | 0.017 | 0.017 | | | | | |
| 10 | rhodopolium_KAlho24IX1980 | 0.015 | 0.015 | 0.015 | 0.016 | 0.019 | 0.018 | 0.018 | 0.018 | 0.000 | | | | |
| 11 | rhodopolium_KK157-14 | 0.015 | 0.015 | 0.015 | 0.016 | 0.019 | 0.017 | 0.017 | 0.017 | 0.000 | 0.000 | | | |
| 12 | rhodopolium_KK1664-12 | 0.015 | 0.015 | 0.015 | 0.016 | 0.019 | 0.017 | 0.017 | 0.017 | 0.000 | 0.000 | 0.000 | | |
| 13 | rhodopolium_neo-KK213-14 | 0.015 | 0.015 | 0.015 | 0.016 | 0.019 | 0.017 | 0.017 | 0.017 | 0.000 | 0.000 | 0.000 | 0.000 | |

1.6 ± 0.1 (%)
(nidorosum vs rhodopolium)

**Figure 3S**. P-distance between the known and new species estimated by MEGA software (*a-d*).
*a*, p-distance between the known species.

## *p*-distance between *E. lacus* and *E. majaloides*

(b)

| | | | E.lacus | | | | | | | | | | E.majaloides | | | | |
|---|---|---|---|---|---|---|---|---|---|---|---|---|---|---|---|---|---|
| | | | 1 | 2 | 3 | 4 | 5 | 6 | 7 | 8 | 9 | 10 | 11 | 12 | 13 | 14 | 15 |
| E. lacus | 1 | KUB106 | | | | | | | | | | | | | | | |
| | 2 | KUB108 | 0.000 | | | | | | | | | | | | | | |
| | 3 | KUB110 | 0.000 | 0.000 | | | | | | | | | | | | | |
| | 4 | KUB113 | 0.000 | 0.000 | 0.000 | | | | | | | | | | | | |
| | 5 | KUB114 | 0.000 | 0.000 | 0.000 | 0.000 | | | | | | | | | | | |
| | 6 | KUB104 | 0.001 | 0.001 | 0.001 | 0.001 | 0.001 | | | | | | | | | | |
| | 7 | KUB107 | 0.001 | 0.001 | 0.001 | 0.001 | 0.001 | 0.000 | | | | | | | | | |
| | 8 | KUB109 | 0.001 | 0.001 | 0.001 | 0.001 | 0.001 | 0.000 | 0.000 | | | | | | | | |
| | 9 | KUB111 | 0.001 | 0.001 | 0.001 | 0.001 | 0.001 | 0.000 | 0.000 | 0.000 | | | | | | | |
| | 10 | KUB105 | 0.001 | 0.001 | 0.001 | 0.001 | 0.001 | 0.000 | 0.000 | 0.000 | 0.000 | | | | | | |
| | 11 | majaloides_KK405-09 | 0.032 | 0.032 | 0.032 | 0.032 | 0.032 | 0.033 | 0.033 | 0.033 | 0.033 | 0.033 | | | | | |
| | 12 | majaloides_KK782-12 | 0.028 | 0.028 | 0.028 | 0.028 | 0.028 | 0.030 | 0.030 | 0.030 | 0.030 | 0.030 | 0.005 | | | | |
| | 13 | majaloides_KK887-06JVauras | 0.030 | 0.030 | 0.030 | 0.030 | 0.030 | 0.031 | 0.031 | 0.031 | 0.031 | 0.031 | 0.006 | 0.001 | | | |
| | 14 | majaloides_KK54-14 | 0.031 | 0.031 | 0.031 | 0.031 | 0.031 | 0.032 | 0.032 | 0.032 | 0.032 | 0.032 | 0.015 | 0.011 | 0.012 | | |
| | 15 | majaloides_KK124-08JVauras | 0.030 | 0.030 | 0.030 | 0.030 | 0.030 | 0.031 | 0.031 | 0.031 | 0.031 | 0.031 | 0.013 | 0.009 | 0.010 | 0.007 | |

3.1 ± 0.1 (%)
(lacus vs majaloides)

**Figure 3S**. P-distance between the known and new species estimated by MEGA software (*a-d*).
*b*, p-distance between *E. lacus* and *E. majaloides*.

# *p*-distance between *E. subrhodopolium, E. sinuatum*, *E. nidorosum*, and *E. rhodopolium*

(c)

| | | *E.subrhodopolium* | | | | | | | |
|---|---|---|---|---|---|---|---|---|---|
| | | 1 | 2 | 3 | 4 | 5 | 6 | 7 | 8 |
| *E. subrhodopolium* | KUB3_seq1 | | | | | | | | |
| | KUB3_seq2 | 0.001 | | | | | | | |
| | KUB123 | 0.001 | 0.000 | | | | | | |
| | KUB130 | 0.001 | 0.000 | 0.000 | | | | | |
| | KUB132 | 0.004 | 0.005 | 0.005 | 0.005 | | | | |
| | KUB1 | 0.010 | 0.005 | 0.005 | 0.005 | 0.006 | | | |
| | KUB124 | 0.010 | 0.005 | 0.005 | 0.005 | 0.006 | 0.000 | | |
| | E_rhodopolium_Er1 | 0.010 | 0.005 | 0.005 | 0.005 | 0.006 | 0.000 | 0.000 | |
| | E_sinuatum_st45 | 0.097 | 0.071 | 0.070 | 0.070 | 0.070 | 0.072 | 0.073 | 0.073 |
| | E_sinuatum_st182 | 0.097 | 0.071 | 0.070 | 0.070 | 0.070 | 0.072 | 0.073 | 0.073 |
| | E_sinuatum_H6003960 | 0.097 | 0.071 | 0.070 | 0.070 | 0.070 | 0.072 | 0.073 | 0.073 |
| | eminens_holoKK417-12 | 0.078 | 0.079 | 0.079 | 0.079 | 0.079 | 0.084 | 0.084 | 0.084 |
| | eminens_KK40-14 | 0.076 | 0.075 | 0.075 | 0.075 | 0.077 | 0.081 | 0.081 | 0.081 |

7.5 ± 0.9 (%)
(subrhodopolium vs sinuatum)

7.9 ± 0.3 (%)
(subrhodopolium vs eminens)

| | | *E.nidorosum* | | | | | | | | *E.rhodopolium* | | | | |
|---|---|---|---|---|---|---|---|---|---|---|---|---|---|---|
| | | 12 | 13 | 14 | 15 | 16 | 17 | 18 | 19 | 20 | 21 | 22 | 23 | 24 |
| *E. subrhodopolium* | KUB3_seq1 | 0.095 | 0.093 | 0.093 | 0.094 | 0.098 | 0.091 | 0.092 | 0.092 | 0.097 | 0.100 | 0.097 | 0.097 | 0.097 |
| | KUB3_seq2 | 0.092 | 0.091 | 0.091 | 0.090 | 0.093 | 0.088 | 0.088 | 0.088 | 0.095 | 0.097 | 0.095 | 0.095 | 0.095 |
| | KUB123 | 0.092 | 0.091 | 0.091 | 0.090 | 0.093 | 0.088 | 0.088 | 0.088 | 0.095 | 0.097 | 0.095 | 0.095 | 0.095 |
| | KUB130 | 0.092 | 0.091 | 0.091 | 0.090 | 0.093 | 0.088 | 0.088 | 0.088 | 0.095 | 0.097 | 0.095 | 0.095 | 0.095 |
| | KUB132 | 0.099 | 0.098 | 0.098 | 0.099 | 0.103 | 0.096 | 0.096 | 0.096 | 0.099 | 0.101 | 0.099 | 0.099 | 0.099 |
| | KUB1 | 0.095 | 0.093 | 0.093 | 0.093 | 0.096 | 0.091 | 0.091 | 0.091 | 0.096 | 0.098 | 0.096 | 0.096 | 0.096 |
| | KUB124 | 0.095 | 0.093 | 0.093 | 0.093 | 0.096 | 0.091 | 0.091 | 0.091 | 0.096 | 0.098 | 0.096 | 0.096 | 0.096 |
| | E_rhodopolium_Er1 | 0.095 | 0.093 | 0.093 | 0.093 | 0.096 | 0.091 | 0.091 | 0.091 | 0.096 | 0.098 | 0.096 | 0.096 | 0.096 |
| | E_sinuatum_st45 | 0.077 | 0.075 | 0.075 | 0.075 | 0.070 | 0.073 | 0.073 | 0.073 | 0.079 | 0.080 | 0.079 | 0.079 | 0.079 |
| | E_sinuatum_st182 | 0.077 | 0.075 | 0.075 | 0.075 | 0.070 | 0.073 | 0.073 | 0.073 | 0.079 | 0.080 | 0.079 | 0.079 | 0.079 |
| | E_sinuatum_H6003960 | 0.077 | 0.075 | 0.075 | 0.075 | 0.070 | 0.073 | 0.073 | 0.073 | 0.079 | 0.080 | 0.079 | 0.079 | 0.079 |

9.3 ± 0.3 (%)
(subrhodopolium vs nidorosum)

9.6 ± 0.2 (%)
(subrhodopolium vs nidorosum)

**Figure 3S**. P-distance between the known and new species estimated by MEGA software (*a-d*).
*c*, p-distance between *E. subrhodopolium, E. sinuatum*, *E. nidorosum*, and *E. rhodopolium*.

## *p*-distance between *E. pseudorhodopolium, E. lupinum*, and *E. rhodopolium*

(d)

| | E.pseudorhodopolium | | | | | | | | | | | | | E.lupium | | E.rhodopolium | | | | |
|---|---|---|---|---|---|---|---|---|---|---|---|---|---|---|---|---|---|---|---|---|
| | 1 | 2 | 3 | 4 | 5 | 6 | 7 | 8 | 9 | 10 | 11 | 12 | 13 | 14 | 15 | 16 | 17 | 18 | 19 | 20 |
| *E. pseudorhodopolium* KUB9 | | | | | | | | | | | | | | | | 0.123 | 0.121 | 0.123 | 0.123 | 0.123 |
| KUB10 | 0.006 | | | | | | | | | | | | | | | 0.123 | 0.124 | 0.123 | 0.123 | 0.123 |
| KUB7 | 0.006 | 0.004 | | | | | | | | | | | | | | 0.119 | 0.121 | 0.119 | 0.119 | 0.119 |
| KUB5 | 0.006 | 0.004 | 0.002 | | | | | | | | | | | | | 0.121 | 0.123 | 0.121 | 0.121 | 0.121 |
| KUB6 | 0.005 | 0.002 | 0.001 | 0.001 | | | | | | | | | | | | 0.119 | 0.121 | 0.119 | 0.119 | 0.119 |
| KUB101_seq1 | 0.005 | 0.002 | 0.001 | 0.001 | 0.000 | | | | | | | | | | | 0.119 | 0.121 | 0.119 | 0.119 | 0.119 |
| KUB101_seq2 | 0.005 | 0.002 | 0.001 | 0.001 | 0.000 | 0.000 | | | | | | | | | | 0.119 | 0.121 | 0.119 | 0.119 | 0.119 |
| KUB102 | 0.005 | 0.002 | 0.001 | 0.001 | 0.000 | 0.000 | 0.000 | | | | | | | | | 0.119 | 0.121 | 0.119 | 0.119 | 0.119 |
| KUB126 | 0.005 | 0.002 | 0.001 | 0.001 | 0.000 | 0.000 | 0.000 | 0.000 | | | | | | | | 0.119 | 0.121 | 0.119 | 0.119 | 0.119 |
| KUB127_seq2 | 0.005 | 0.002 | 0.001 | 0.001 | 0.000 | 0.000 | 0.000 | 0.000 | 0.000 | | | | | | | 0.119 | 0.121 | 0.119 | 0.119 | 0.119 |
| KUB128 | 0.005 | 0.002 | 0.001 | 0.001 | 0.000 | 0.000 | 0.000 | 0.000 | 0.000 | 0.000 | | | | | | 0.119 | 0.121 | 0.119 | 0.119 | 0.119 |
| ectomycorrhizal_P09083 | 0.005 | 0.002 | 0.001 | 0.001 | 0.000 | 0.000 | 0.000 | 0.000 | 0.000 | 0.000 | 0.000 | | | | | 0.119 | 0.121 | 0.119 | 0.119 | 0.119 |
| KUB127_seq1 | 0.007 | 0.005 | 0.004 | 0.002 | 0.002 | 0.002 | 0.002 | 0.002 | 0.002 | 0.002 | 0.002 | 0.002 | | | | 0.121 | 0.123 | 0.121 | 0.121 | 0.121 |
| lupinum_holoKK13-14JVauras | 0.052 | 0.051 | 0.047 | 0.050 | 0.049 | 0.049 | 0.049 | 0.049 | 0.049 | 0.049 | 0.049 | 0.049 | 0.049 | | | 0.101 | 0.103 | 0.101 | 0.101 | 0.101 |
| lupinum_126-05 | 0.053 | 0.053 | 0.048 | 0.050 | 0.049 | 0.049 | 0.049 | 0.049 | 0.049 | 0.049 | 0.049 | 0.049 | 0.049 | 0.001 | | 0.101 | 0.103 | 0.101 | 0.101 | 0.101 |

4.9 ± 0.1 (%)
(pseudorhodopolium vs lupinum)

10.1 ± 0.1 (%)
(pseudorhodopolium vs rhodopolium)

**Figure 3S**. P-distance between the known and new species estimated by MEGA software (*a-d*).
*d*, p-distance between *E. pseudorhodopolium, E. lupinum*, and *E. rhodopolium*.

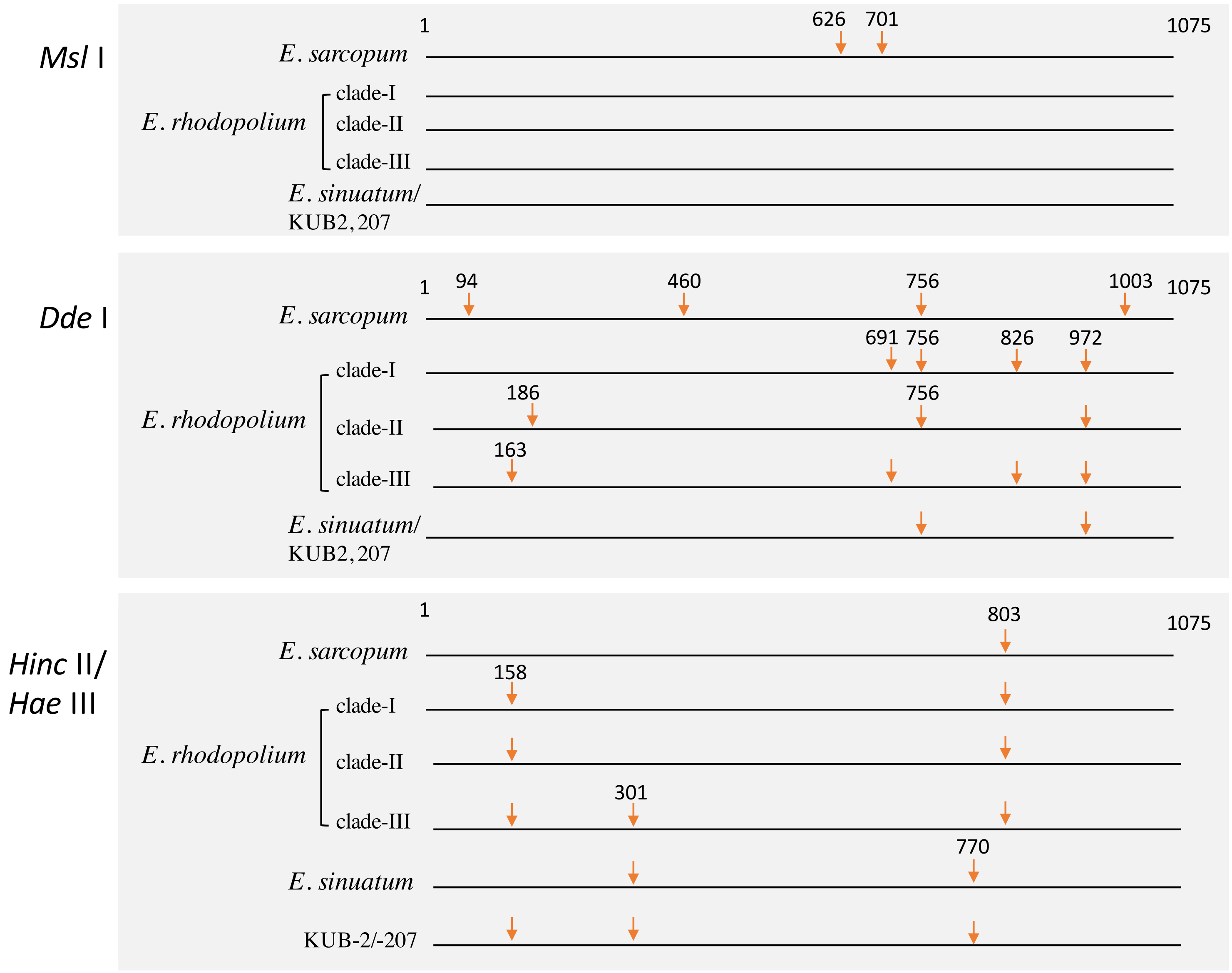


**Figure 4S** Restriction endonucleases were used for discrimination between edible and poisonous mushrooms and for species classification. MslI digestion revealed the presence of edible E. sarcopum. Other Entoloma species were not digested. DdeI and a combination of HincII/HaeIII double digestion provide different band pattern between three clades of Japanese E. rhodopolium.

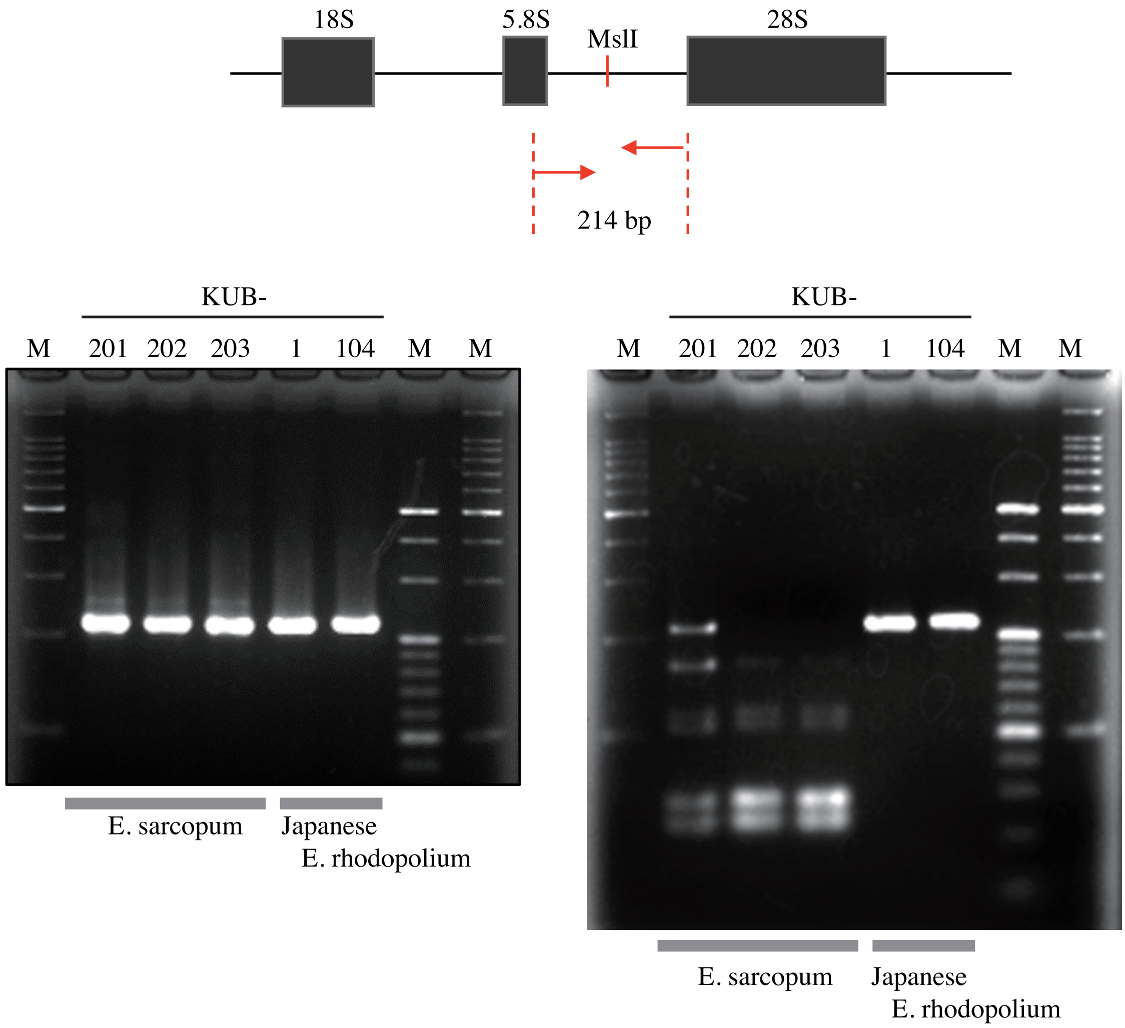


**Figure 5S.** Restriction endonucleases were used for discrimination between edible and poisonous mushrooms and for species classification. MslI digestion revealed the presence of edible E. sarcopum. Other *Entoloma* species were not digested. DdeI and a combination of HincII/HaeIII double digestion provide different band pattern between three clades of Japanese *E. rhodopolium*.

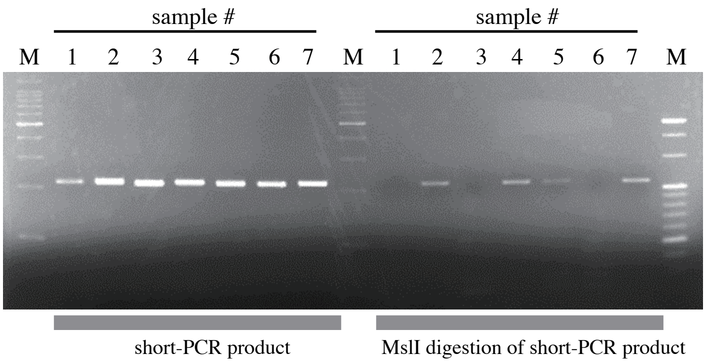


quasi-mixed mushroom

| sample | edible | *E. subrhodopolium* | *E. sarcopum* |
|---|---|---|---|
| 1 | 400 | − | − |
| 2 | − | 200 | − |
| 3 | − | − | 200 |
| 4 | 200 | 200 | − |
| 5 | 200 | 100 | 100 |
| 6 | 200 | − | 200 |
| 7 | 380 | 20 | − |

**Figure 6S.** Detection of poisonous Japanese *E. rhodopolium* clades in quasi-mixed mushroom samples. A, content of each sample. B, band patterns on 2% agarose gel of samples 1 to 7. PCR products (214 bp) in samples 2, 4, 5, and 7 were left intact, whereas samples 1, 3, and 5 were completely digested. Experimental conditions were the same as that in Figure.6.

Samples 2, 4, 5, and 7 contain poisonous *E. subrhodopolium*.
